# Prevalence of Mastitis and Antibiotic Resistance of Bacterial Isolates from CMT Positive Milk Samples Obtained from Dairy Cows, Camels, and Goats in Two Pastoral Districts in Southern Ethiopia

**DOI:** 10.3390/ani11061530

**Published:** 2021-05-24

**Authors:** Amanuel Balemi, Balako Gumi, Kebede Amenu, Sisay Girma, Mu'uz Gebru, Muluken Tekle, Agustin A. Ríus, Doris H. D’Souza, Getahun E. Agga, Oudessa Kerro Dego

**Affiliations:** 1 College of Veterinary Sciences, Mekelle University, Mekelle, P.O. Box 231, Ethiopia; balemi2005@gmail.com (A.B.); muuz.gebru@mu.edu.et (M.G.); 2 Aklilu Lemma Institute of Pathobiology, Addis Ababa University, Addis Ababa, P.O. Box 1176, Ethiopia; balako.gumi@yahoo.com; 3 College of Veterinary Medicine and Agriculture, Addis Ababa University, Bishoftu, P.O. Box 34, Ethiopia; kamenu@gmail.com (K.A.); mulukentekle@gmail.com (M.T.); 4 College of Agriculture, Bule Hora University, Bule Hora, P.O. Box 144, Ethiopia; sisaygirma80@gmail.com; 5 Department of Animal Science, The University of Tennessee, Knoxville, TN 37996, USA; arius@utk.edu; 6 Department of Food Science, The University of Tennessee, Knoxville, TN 37998, USA; ddsouza@utk.edu; 7 Food Animal Environmental Systems Research Unit, Agricultural Research Service, U.S. Department of Agriculture, Bowling Green, KY 42101, USA; getahun.agga@usda.gov

**Keywords:** antimicrobial resistance, mastitis, prevalence, dairy cow, dairy camel, dairy goat, bacterial mastitis pathogen

## Abstract

**Simple Summary:**

A study was carried out from August 2017 to February 2018 on lactating dairy cows, one-humped dromedary camels, and goats to determine mastitis in the Bule Hora and Dugda Dawa districts of in Southern Ethiopia. Milk samples from 564 udder quarters and udder halves from 171 animals consisting of 60 dairy cows, 51 camels, and 60 goats were tested for mastitis. Sixty-four positive udder milk samples were cultured, and bacterial mastitis pathogens were isolated and identified. The antibiotic resistance of bacterial isolates from milk with mastitis was tested against nine antimicrobials commonly used in the study area. Cow-level prevalence of mastitis in dairy cows, camels, and goats was 33.3%, 26.3%, and 25%, respectively. The quarter-level prevalence of mastitis in cows, camels and goats was 17.6%, 14.5%, and 20%, respectively. In cattle, the prevalence was significantly higher in Dugda Dawa than in Bule Hora. Major bacterial isolates were coagulase-negative *Staphylococcus* species (39.1%), *S. aureus* (17.2%), *S. hyicus* (14.1%), and *S. intermedius* and *Escherichia coli* (9.4% each). In camels, udder abnormality and mastitis were significantly higher in late lactation than in early lactation. Mastitis tends to increase with parity in camels. *E. coli* isolates were highly resistant to spectinomycin, vancomycin, and doxycycline, whereas most *S. aureus* isolates were multidrug-resistant. Most of the rural and periurban communities in this area consume raw milk, which indicates a high risk of infection with multidrug-resistant bacteria. We recommend community-focused training programs to improve community awareness of the risk of raw milk consumption and the need to boil milk.

**Abstract:**

A study was carried out from August 2017 to February 2018 on lactating dairy cows, one-humped dromedary camels, and goats to determine mastitis in the Bule Hora and Dugda Dawa districts of in Southern Ethiopia. Milk samples from 564 udder quarters and udder halves from 171 animals consisting of 60 dairy cows, 51 camels, and 60 goats were tested for mastitis. Sixty-four positive udder milk samples were cultured, and bacterial mastitis pathogens were isolated and identified. The antibiotic resistance of bacterial isolates from milk with mastitis was tested against nine antimicrobials commonly used in the study area. Cow- and quarter-level prevalence of mastitis in dairy cows, camels, and goats was 33.3%, 26.3%, and 25% and 17.6%, 14.5%, and 20%, respectively. In cattle, the prevalence was significantly higher in Dugda Dawa than in Bule Hora. Major bacterial isolates were coagulase-negative *Staphylococcus* species (39.1%), *S. aureus* (17.2%), *S. hyicus* (14.1%), and *S. intermedius* and *Escherichia coli* (9.4% each). In camels, udder abnormality and mastitis were significantly higher in late lactation than in early lactation. Mastitis tends to increase with parity in camels. *E. coli* isolates were highly resistant to spectinomycin, vancomycin, and doxycycline, whereas most *S. aureus* isolates were multidrug-resistant. Most of the rural and periurban communities in this area consume raw milk, which indicates a high risk of infection with multidrug-resistant bacteria. We recommend a community-focused training program to improve community awareness of the need to boil milk and the risk of raw milk consumption.

## 1. Introduction

Mastitis, an inflammation of the mammary gland caused by an infection, trauma, or injury to the udder, is one of the most common diseases of dairy animals that affects the wellbeing of livestock populations in this study area [1,2,3,4,5,6,7]. Mastitis causes substantial economic losses due to reduced milk yield, treatment costs, discarding of milk with antibiotics, the lower price of poor-quality milk, and death from severe inflammation [8,9]. In Ethiopia, mastitis causes major economic losses, mainly due to milk production losses and culling [10,11,12]. About 137 infectious agents are known to cause mastitis in large domestic animals, of which bacteria are the major ones [13]. The most common bacteria that cause mastitis are *Staphylococcus*, *Streptococcus,* and coliform bacteria (*E. coli*, *Klebsiella* spp., and *Enterobacter* spp.) [14]. *Staphylococcus aureus* is one of the leading causes of mastitis in dairy cattle in Ethiopia [3,15,16,17], resulting in significant economic losses due to direct and indirect costs [10]. Similarly, *Streptococcus* spp. and coliform bacteria are frequently reported to cause mastitis in Ethiopia [3,5,17,18]. 

*Staphylococcus aureus* is a contagious mastitis pathogen. *Staphylococcus aureus* is a Gram-positive, catalase and coagulase-positive, non-spore-forming, oxidase negative, non-motile, cluster-forming, facultative anaerobe [19]. *Staphylococcus aureus is* usually isolated from different body parts of dairy animals, including the head, skin, leg, and nasal mucosa as well as the milker’s hands. However, an infected udder quarter remains the main reservoir of infection for non-infected animals during the milking time through contaminated milkers’ hands, towels, and milking machines [20]. *Staphylococcus aureus* can be distinguished from other staphylococcal species on the basis of the production of coagulase, the fermentation of mannitol, and trehalose [21]. The coagulase test is not an absolute test for confirming the diagnosis of *S. aureus* in cases of bovine mastitis; however, more than 95% of all coagulase-positive staphylococci from bovine mastitis belong to *S. aureus* [22].

Coliform bacteria include the genera *Escherichia, Klebsiella*, and *Enterobacter* [23]. Coliform bacteria are a major cause of clinical mastitis [24]. The most common species, isolated in more than 80% of cases of coliform mastitis, is *Escherichia coli* [25,26]. *E. coli* usually infects the mammary glands during the dry period and progresses to inflammation and clinical mastitis during early lactation, with local and sometimes severe systemic clinical manifestations. If the infection is localized in the mammary gland and there is no systemic involvement, treatment with an antibiotic is not recommended, since it worsens the inflammatory response due to bacterial death and the release of LPS, which might lead to poor prognosis and worse animal welfare. Clinical mastitis may display severe systemic clinical manifestations. For example, there are reports that found 32% of coliform mastitis cases showed bacteremia (the presence of bacteria in the circulating blood) [27,28]. Approximately 10% of clinical mastitis may lead to death [29].

In general, previously published data did not demonstrate widespread antimicrobial resistance (AMR) among mastitis pathogens [30,31]. However, recent studies have shown increased resistance against tetracycline among *S. aureus* [32] and *E.* *coli* isolates [33] from cases of mastitis. There is no doubt that antimicrobial usage in food animal production leads to an increase in AMR [34,35]. Dairy farms may serve as a source of antimicrobial-resistant human pathogenic bacteria, especially extended spectrum beta-lactamases producing *E. coli* [33,36] and colistin-resistant *E. coli* [37]. Extensive use of third-generation cephalosporins (3GCs) in dairy cattle for the prevention and treatment of mastitis [31,38,39] and other diseases of dairy cattle [40,41] could result in the carriage of extended-spectrum beta-lactamase-producing *Enterobacteriaceae* (ESBL Ent) [33,42,43]. 

The somatic cell counts (SCCs) of milk from dairy cattle, camels, and goats are different but the California Mastitis Test (CMT) has been used frequently to estimate increased somatic cell counts in these species. SCC has been determined in camel milk to diagnose clinical or subclinical mastitis [44,45,46]. The SCC values in the bulk milk of clinically healthy dromedaries are higher than those from dairy cows but lower than those in sheep and goats [47]. Similarly, SCC is used to determine subclinical mastitis in goats [48]. Generally, healthy goats have a higher milk SCC compared with sheep and other ruminants such as cows. Some have reported a SCC  of ≥ 10^6^ cells/mL as an indication of subclinical mastitis in goats; however, this set minimum is usually combined with a bacteriological test to confirm diagnosis. The SCC with a bacteriological test is the most reliable indicator of subclinical mastitis in goats [49]. In addition, the SCC in goat milk varies based on the stage of lactation, and it has been reported to reach 3.6 × 10^6^ cells/mL at the end of lactation [50].

The interaction between milk quality parameters and various factors influencing milk quality has been studied extensively in conventional dairy species. In cow and sheep milk, low bulk tank bacterial count is associated with low SCC and the increase in one parameter coincides with the increase of the other [51,52,53]. Milk quality parameters are influenced by many factors, including year, season, month, herd, age, parity, breed, stage of lactation, intramammary infection, environmental factors, and management practices [54,55,56,57,58,59,60]. The complex interactions among the abovementioned factors determine the final quality of the bulk tank milk. In the United States, the pyronin Y-methyl green (PMG) staining procedure is considered the standard confirmatory test and is the official reference method for direct microscopic somatic cell count (DMSCC) in goat milk [54]. A similar method has been adopted for SCC in camel milk in other areas [47].

This study area is close to the border between Ethiopia and Kenya, and indiscriminate use of antimicrobial drugs from different sources to treat animal and human diseases is very common. Raw milk consumption is a widely practiced culture in this area, which predisposes the consumers to milk-borne infections. The objectives of this study were (1) to determine the prevalence of mastitis in dairy cattle, goats, and camels in this study area and (2) to isolate and identify the etiological agents from cases of mastitis and determine the antibiotic resistance patterns of bacterial isolates against commonly used antibiotics in the area.

## 2. Materials and Methods

### 2.1. Study Setting and Sampling

**Figure 1:**
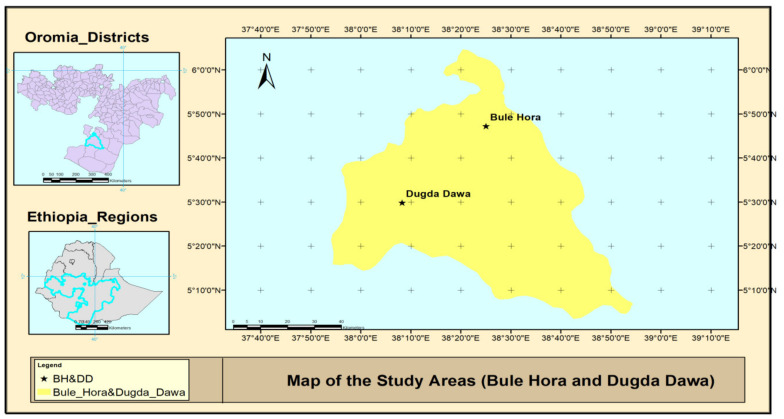
A map showing the locations of the study areas: the Hule Hora and Dugda Dawa districts of the West Guji Zone, Oromia State, Ethiopia.

A cross-sectional study was conducted from September 2017 to May 2018 in the Bule Hora and Dugda Dawa districts of the West Guji Zone of Oromia, Ethiopia (Figure 1). West Guji Zone is characterized mainly by the pastoral animal production system, and the livelihood of the pastoral communities depends on their animals. Unlike the sedentary production system in the mid-highlands and highlands of Ethiopia, where cattle are the major dairy animals, camels and goats are also reared for milk production in the lowlands of Ethiopia. While the Dugda Dawa district is in the lowland (1500 m above sea level) pastoral area, the Bule Hora district is in the mid-highland (1500–1800 m above sea level) agro-pastoral ecological zone. A total of 171 lactating local indigenous breed dairy cows (*n* = 60), dromedary camels (*n* = 51), and dairy goats (*n* = 60) that were kept under traditional management systems were selected for this study. The study areas were selected based on the availability of dairy cows, camels, and goats and accessibility to the area by car. A systematic random sampling technique in which households (referred to as herds) were first randomly selected, followed by a random selection of the study animals within each herd.

### 2.2. Physical Clinical Examination, Milk Collection, and California Mastitis Testing (CMT) 

Milk samples were collected early in the morning or late in the afternoon at the time of milking. Animals selected for the study were clinically examined for any visible abnormality on each udder quarter or udder half at the time of sample collection. Visible inflammatory abnormalities such as swelling, pain, heat, redness on each udder unit (each cow or camel has four udder quarters/units, and each goat has two udder halves/units) were scored. Scores were recorded as 0 (no change and normal appearance), 1 (slight swelling in the udder, 2 (moderate swelling and pain in the udder, 3 (severe swelling, pain, heat, and/or hardness of the udder detected), and 4 (nonfunctional blind udder). Udder quarters of cows and camels or udder halves of goats were washed and dried, and the teat openings were scrubbed with 70% alcohol prior to sample collection. Milk samples were aseptically collected into sterile universal bottles from each quarter or each udder half after discarding the first 2–3 squirts of milk. For each animal, parity number, stage of lactation, any visible lesions on the udder, and milk appearance were recorded. Milk samples were also examined for any visible inflammatory abnormalities such as change of color, viscosity, and the presence of flakes and clots. Milk appearance was scored as 0 (normal milk appearance), 1 (watery appearance), 2 (flakes in the milk), and 3 (clots, color change or bad odor). The milk samples were kept on ice in a cooler box and transported to the Microbiology Laboratory at the College of Veterinary Medicine and Agriculture of Addis Ababa University in Bishoftu, Oromia.

The somatic cell counts in milk from dairy cattle, camels, and goats are different. There is no standardized method to determine the somatic cell count in the milk of each of these dairy species that is widely available to use. Therefore, we used the California Mastitis Test (CMT), which is an indirect quick test to estimate somatic cell count. Individual udder milk samples from udder quarters and udder halves were analyzed by CMT for detecting subclinical mastitis. The CMT test was performed and interpreted as described by Schalm and Noorlander [61]. Briefly, 2 mL of milk was added into each chamber of the CMT paddle. An equal volume of CMT reagent (ImmuCell Corporation, Portland, OR, USA) was added to each chamber and mixed thoroughly by gently rotating the paddle in a circular motion in a horizontal plane for 10 s at ambient temperature. The CMT reagent causes lysis of somatic cells, releasing DNA and proteins, which increase the viscosity of the mixture. An increase in the viscosity is an indication of the increased somatic cell count, and the result is visually scored using a 5-point score as 0 (negative, mixture remain liquid with no visible precipitation), trace (a slight precipitate which tends to disappear with continued movement of the paddle), positive 1+ (mild reaction, a distinct precipitate but not forming a gel), positive 2+ (moderate reaction, the mixture thickens immediately with some gel formation), and positive 3+ (strong reaction, a distinct gel that adheres to the bottom of the paddle formed). Milk samples with CMT scores of 0 and trace were recorded as negative. In this study, clinical mastitis was defined as an udder quarter or udder half with visible abnormal inflammatory changes in the mammary gland tissue such as redness, swelling, pain, or increased heat and/or visible inflammatory changes in the milk such as a change in color (watery, bloody, blood-tinged, serum-like, etc.) or a change in consistency (clots or flakes, or stringy or viscous). Subclinical mastitis was defined as an udder quarter or an udder half with a CMT score of ≥1+. CMT scores were reclassified as present/absent to define the prevalence of mastitis on the udder quarter or udder half basis.

### 2.3. Bacteriological Culture, Isolation, and Identification of Bacteria from CMT-Positive Samples

Bacteriological culture, isolation, and identification of the bacterial causative agent were performed for 64 milk samples that had CMT scores of 2+ or 3+ following the National Mastitis Council guidelines [62]. Briefly, a loopful (10 μL) of milk sample was inoculated into tryptic soy agar with 5% sheep blood (blood agar plates) (Oxoid, Basingstoke, UK) and incubated at 37 °C for 24–48 h. Plates were evaluated for the growth of bacteria at 24 and 48 h of incubation, and colony characteristics, pigment production, and hemolysis were recorded. The individual culture was sub-cultured onto nutrient agar plates (Oxoid, Basingstoke, UK) to get a pure colony. One colony each per pure culture was Gram-stained and differentiated into Gram-positive or Gram-negative bacteria. Gram-positive cocci were further tested with a catalase test to differentiate *Staphylococcus* species from *Streptococcus* species. Catalase-positive cocci were considered to be *Staphylococcus* species and further tested for mannitol fermentation. Mannitol-positive isolates were tested by the tube coagulase test using rabbit plasma to differentiate *S. aureus* from other coagulase-negative *Staphylococcus* species. Coagulase-positive *Staphylococcus* species were further tested with the Voge–Proskauer (VP), trehalose fermentation, and O-nitrophenyl-beta-D-galactopyranoside (ONGP) tests to differentiate *S. aureus*, *S. hyicus*, and *S. intermedius*. The oxidase test was used to differentiate the *Enterobacteriaceae* from Gram-negative non-*Enterobacteriaceae* organisms. *Enterobacteriaceae* are oxidase-negative. Oxidase-negative members of *Enterobacteriaceae* were further inoculated into MacConkey agar (Oxoid) and analyzed by biochemical tests. The biochemical tests used included lactose fermentation, indole production, the methyl red test, Voge–Proskauer, citrate utilization, hydrogen sulfide in TSI agar, lysine decarboxylase, and urease activity. Each pure bacterial isolate was inoculated into tryptic soy broth (TSB) (Oxoid) and grown overnight at 37 °C, and 500 μL of the overnight culture (16–18 h) was mixed with 500 μL of sterile 85% glycerol and stored at −80 °C for additional tests.

### 2.4. Antimicrobial Susceptibility Testing

Antimicrobial susceptibility testing was conducted for 32 bacterial isolates consisting of *S. aureus*, *S. intermedius*, *S. hyicus*, and *E. coli* obtained from CMT-positive milk samples using the Kirby–Bauer disc diffusion method on Mueller–Hinton agar plates (Oxoid), as described elsewhere [63]. The isolates were tested against a panel of 9 antimicrobials (Oxoid), which included penicillin G (P: 1U), spectinomycin (S: 300 µg), nitrofurantoin (F: 50 µg), polymyxin B (PB: 300 U), vancomycin (VA: 5 µg), ceftriaxone (CRO: 30 µg), chloramphenicol (C: 30 µg), doxycycline (DO: 5 µg), and clindamycin (DA: 10 µg). For each isolate, the inhibition zone diameter was measured to the nearest millimeter, and the result was interpreted as susceptible (S), intermediate (I), or resistant (R) according to the Clinical Laboratories Standards Institute guidelines [64]. Intermediate results were categorized as resistant for this study as per the CLSI guidelines [64]. Isolates resistant to ≥3 antimicrobial classes were considered multidrug-resistant. 

### 2.5. Statistical Analysis

Descriptions of the study animals or samples were given as the frequency or summary mean. Data were analyzed at the level of udder quarter or udder half sample results. The effects of stage of lactation, district, and parity on the prevalence of udder and milk abnormalities, mastitis, and clinical and subclinical mastitis were evaluated after re-categorizing them as present or absent outcomes. We used multilevel mixed-effects logistic regression to account for the clustering of animals within a herd and clustering of udder quarters/halves within an animal. Data were analyzed separately for each animal species. Stage of lactation and district, considered as categorical variables, and parity, considered as a continuous variable, were included in the models as fixed effects. Herd and animal identifiers were included into the models as random effects. Age was excluded due to multicollinearity, since age and parity are correlated. All analyses were conducted in STATA 16.1 (Stata Corp LLC, College Station, TX, USA). 

## 3. Results

### 3.1. Prevalence of Udder and Milk Abnormalities, and Clinical and Subclinical Mastitis in Dairy Cows, Camels, and Goats

In this study, we examined 564 individual udder quarters or udder halves from 60 cows, 51 camels, and 60 goats sampled from 68 herds in two pastoral districts. Mixed species were sampled from four herds: one herd had cattle and camels sampled, two herds had cattle and goats sampled, and one herd had all three animal species sampled. While most of the udder quarters examined were from cows and camels in mid-lactation, most of the udder halves examined in goats were from early lactation (Table 1). 

Over 80% of the udder quarters or udder halves of each animal species were normal for all the outcome parameters assessed (Table 2). Milk samples obtained from the udder quarters or udder halves of cows, camels, and goats had similar proportions of CMT scores (Table 2). As shown in Table 3, 11.3% and 11.8% of the udder quarters in cows and camels, respectively, and 14.2% of the udder halves in goats had some form of abnormality. Milk from 5.6% and 7% of the udder quarters in cows and camels, respectively, and 7.6% of the udder halves in goats had some form of abnormal appearance (Table 3). Based on physical clinical examination of the udder, the milk or CMT scores of each udder quarter and udder half were classified into normal, clinical, subclinical, or blind/nonfunctional. Accordingly, 9% and 10% of the udder quarters in cows and camels, respectively, and 10% of the udder halves in goats had clinical mastitis. Subclinical mastitis was observed in 8.6% and 4.5% of the udder quarters in cows and camels, respectively, and in 10% of the udder halves in goats (Table 3). In camels, the prevalence of udder abnormality (*p* = 0.033) and mastitis (*p* = 0.013) were significantly higher in late lactation than in early lactation. The prevalence of mastitis tended to increase (*p* = 0.028) with parity in camels (Table 4). In cattle, the prevalence of mastitis was significantly (*p* = 0.034) higher in the Dugda Dawa district compared with the Bule Hora district (Table 5). In goats, the prevalence of udder and milk abnormalities, overall mastitis prevalence, and clinical and subclinical mastitis were not affected by stage of lactation, district, and parity (Table 6). The prevalence of mastitis in dairy cows, camels, and goats was 33.3%, 26.3%, and 25%, respectively. In dairy cattle, the cow- and quarter-level prevalence was 33.3% and 17.1%, respectively. Cow-level prevalence of clinical and subclinical mastitis constituted 15% and 18.3%, respectively. The overall prevalence of mastitis in dairy camels was 29.4% and 14.2% at the animal and quarter level, respectively. Of the 29.4%, clinical and subclinical mastitis constituted 9.8% and 19.6%, respectively. The prevalence of mastitis in goats was 25%, which comprised 10% and 15% clinical and subclinical mastitis, respectively.

#### 3.1.1. Bacteria Isolated from Milk Samples

From 64 milk samples with CMT scores of ≥ 2+, cultured bacteria were obtained from 62 (97%%) of the samples. The bacterial species identified across the 64 milk samples tested were coagulase-negative *Staphylococcus* spp. (CNS) (39.1%), *Staphylococcus aureus* (17.2%), *Staphylococcus hyicus* (14.1%), and *Staphylococcus intermedius* and *E. coli* (9.4% each) (Table 7).

#### 3.1.2. Antimicrobial Resistance of Bacteria Isolated from Milk

The highest resistance was observed to penicillin (100%; *n* = 32), followed by spectinomycin (97%), ceftriaxone (82%), clindamycin (75%), and vancomycin (66%), as shown in Table 8. All *E. coli* isolates, from both cows and goats, were resistant to spectinomycin, vancomycin, ceftriaxone, and doxycycline. Gram-negative bacteria have an intrinsic resistance against penicillin, so penicillin is not effective against *E. coli*. All *S. aureus* isolates were resistant to penicillin G, spectinomycin, and clindamycin regardless of animal species. All isolates were MDR if they were resistant to more than three antimicrobial classes. Two *E. coli* isolates obtained from cattle were resistant to all nine drugs tested but resistance to penicillin is intrinsic and should not be considered acquired.

## 4. Discussion

Our mastitis prevalence results in dairy cattle were closely similar to those of Haftu et al. [65], who reported a cow-level prevalence of 37.4%, consisting of 3.6% clinical and 33.8% subclinical cases, with a quarter-level prevalence of 17.8% in Northern Ethiopia. Our results were higher than that of Abera et al. [2], who reported a prevalence of 30.3% and 10.3% at the cow and quarter levels, respectively, in small dairy farms in and around Hawassa in Southern Ethiopia. We found a lower prevalence than Lakew et al. [5], Birhanu et al. [66], Abebe et al. [67], Mekonnen et al. [16], Sarba et al. [68], Zeryehun et al. [69], Tolosa et al. [70], Lakew et al. [71], Abdella et al. [17], and Kerro Dego et al. [3] in different parts of Ethiopia. Similarly, Getaneh et al. [4] conducted a meta-analysis of 39 published articles from 2002 to 2016; they found a higher pooled prevalence of 47% at cow level, of which 8.3% and 37% were clinical and subclinical mastitis, respectively. Getahun et al. [18] also reported a high prevalence of 54.7%, 22.3%, and 10.1% of subclinical mastitis and a low prevalence of 8.3%, 1.8%, and 0.51% of clinical mastitis at the herd, cow, and quarter levels, respectively, in crossbreed lactating cows from smallholder dairy farms in the Sellalle area of Central Ethiopia. These variations are mainly because of differences in the production system (intensive, semi-intensive, and extensive), ecology, management, and methodological differences among these studies.

Our mastitis prevalence results in camels were closely similar to those of Abera et al. [72], who reported an overall prevalence of 29% and 17.9% at the animal and quarter levels, respectively, in lactating camels in the Jijiga area of Somali Regional State in Eastern Ethiopia. Our results were lower than those of Regassa et al. [73], who reported a prevalence of 44.8% in camels in the neighboring Borena Zone, of which clinical and subclinical mastitis made up 5.4% and 39.4%, respectively. We found a quarter-level prevalence of 14.6% in camels, which was lower than 24% reported by Regassa et al. [73] in camels in the Borena Zone. These variations may be due to a slight difference in the ecological area and management differences. Similarly, Bekele et al. [7] reported a higher clinical mastitis prevalence of 12.5% in camels from the Afar Region, and Abdel Gadir Afit et al. [1] also reported an intramammary infection (IMI) rate of 59.7%, of which 75% and 25% had major and minor mastitis pathogens, respectively, in camels in the Negele Borena, Dire Dawa, and Gewane areas of Ethiopia. Despite the similarity in the pastoral production systems in these areas, there are several differences in management and mastitis control practices among camel herders in these areas, which might contribute to differences in disease prevalence in these areas.

Our mastitis prevalence results in goats were higher than those of Megersa et al. [6], who reported an overall prevalence of 15.5%, of which clinical and subclinical cases made up 4.3% and 11.2%, respectively, in lactating dairy goats under the same pastoral management system in the neighboring Borena Zone. Despite the similarity of the pastoral production systems, flock management still varies greatly, which might contribute to the observed difference in the prevalence of mastitis. For example, Borana’s pastoral community is in the lowlands, where drought and feed and water shortages are major problems, whereas Guji’s pastoral community in the Bule Hora district is in the mid-highlands, where drought is not a significant problem. Similarly, Dugda Dawa is located in the lowlands but has no water shortage because of proximity to good water sources. Not all pastoralists manage their animals in the same manner: some are good at vaccinating all their animals, whereas some do not vaccinate all animals, which might influence the overall health of each animal.

Mastitis is a complex multifactorial disease involving interactions of various factors such as the type of management and husbandry, environmental conditions, animal risk factors, and causative agent-related factors, so variations in prevalence could be due to variations of these different factors. 

We found 3.3%, 2.5%, and 1.7% blind quarters/halves in cows, camels, and goats, respectively. Lakew et al. [5] reported the same result of 3.3% blind quarters in cows from Haramaya. Our result was lower than that of Sarba et al. [68], Zeryehun et al. [69], and Tolosa et al. [74], who reported blind quarters in 5.5%, 6.6%, and 6% of cows in the Ambo district of the East Shewa Zone, Eastern Hararge Zone, and Jimma, respectively. We found a lower number of blind quarters (2.5%) in camels compared with Abera et al. [72], who reported 33.8% blind quarters in camels from the Jijiga area of Somali Regional State. This variation may be due to differences in treatment against mastitis in camels and health care for animals. 

There were no significant differences in the prevalence of mastitis among different ages, parity number, and stages of lactation in camels, cows, and goats. However, the prevalence of mastitis tended to increase (*p* = 0.028) with parity in camels. Abera et al. [72] also reported that the prevalence of mastitis in camels was significantly affected by tick infestation, udder lesions, increased age, and parity of animals. Our results disagree with a previous study by Regassa et al. [73] in camels, who reported a significantly higher prevalence in early lactation than in late lactation in the Borana Zone. In cows, the prevalence of mastitis was significantly (*p* = 0.034) higher in the Dugda Dawa district compared with the Bule Hora district. Our results agree with that of Abera et al. [2] in cows in Hawassa, who reported no association of the prevalence of mastitis with age, parity, and history of mastitis. On the contrary, others reported a higher prevalence of mastitis in older cows [4,5,65,66,68], in crossbreeds than in indigenous zebu [3,5,67,68,75], in cows at the late lactation stage [2,4,16,18,65,67,74,75], in cows at the early stage of lactation [3,4], in cows with a high parity number [5,16,66,67,68,75], and in cows with teat lesions and/or tick infestations [3,74]. In goats, the prevalence of udder and milk abnormalities, overall mastitis prevalence, and clinical and subclinical mastitis were not affected by the stage of lactation, district, and parity. Our results disagree with that of Megersa et al. [6], who reported that does in the late lactation stage, those with long teats, those with poor body condition, and those examined in the wet season were at high risk of udder infection than those in early lactation, those with short teats, those with good body condition, and those examined in the dry period, respectively. However, significant variations were not observed for udder tick infestation, mixing goats with sheep, and flock size.

These variations might be due to several factors, including study methodology, differences in the number of animals included in the study, managemental differences, ecological differences, and differences in mastitis treatment and control practices among producers and herders.

The most prevalent isolates were coagulase-negative *Staphylococcus* spp. (CNS) (*n* = 25, 39.1%), *Staphylococcus aureus* (*n* = 11, 17.2%), *Staphylococcus hyicus* (*n* = 9, 14.1%), and *Staphylococcus intermedius* and *Escherichia coli* (both *n* = 6, 9.4%). Our results were comparable with the previous report by Regassa et al. [73], who reported the highest prevalence of *S. aureus* at the animal and quarter levels of 12.8% and 2.9%, respectively, in camels in the Borana Zone. Similarly, Mekonnen et al. [16] also reported that the predominant isolates were coagulase-negative *Staphylococcus* spp. (31%), followed by *Staphylococcus aureus* (9%) in cattle. Haftu et al. [65] also reported *S. aureus* (36%) and *E. coli* (27.3%) as the major isolates from cases of mastitis in dairy cattle. Abebe et al. [67] reported that *S. aureus* was isolated from 51.2% of milk samples cultured and 73.2% of the herd affected with mastitis. Tolosa et al. [74] reported that non-*aureus* staphylococci were the most frequently isolated pathogens in both clinical mastitis cases and IMI. Different bacterial etiological agents were reported by different authors, including Bekele et al. [7], Abera et al. [2], Abdella et al. [17], Almaw et al. [75], Birhanu et al. [66], Getahun et al. [18], and Lakew et al. [71], which mainly included *S. aureus*, coagulase-negative staphylococci, *Streptococcus agalactiae*, *Streptococcus dysgalactiae*, *Streptococcus uberis*, other *Streptococcus* spp., *Bacillus* spp, *Pasteurella hemolytica,* and *E. coli* [7]. 

The high prevalence of *S. aureus* in this study might be associated with the absence of hygienic milking practices, a lack of culling of cows chronically infected with *S. aureus*, and consistent hand-milking practices throughout the dairy herds. Since *S. aureus* is usually found on the udder or teat skin surface of infected animals, the primary source of transmission from infected udders to uninfected is usually by the milkers’ hands during hand-milking.

*S. aureus* isolates in this study showed high sensitivity to vancomycin, doxycycline, and ceftriaxone. This might be due to limited usage of these antimicrobials for the treatment of diseases of these species of dairy animals, including mastitis. This study showed that all *S. aureus* isolates from cows, camels, and goats were resistant to penicillin G (100%) and spectinomycin (100%). Overall, we found that all *S. aureus* isolates were multidrug-resistant, which agrees with the study of Haftu et al. [65], who reported that all *S. aureus* isolates from cows were multidrug-resistant, that were resistant to ampicillin, erythromycin, clindamycin, and chloramphenicol. This resistance might be due to repeated therapeutic and/or indiscriminate use of these antimicrobials in these study areas. 

*S. aureus* isolates from cows were resistant to penicillin G (100%), spectinomycin (100%), clindamycin (83.33%), and vancomycin (83.33%). These results were in agreement with reports from [76] in and around Assosa that suggested a possible development of resistance from prolonged and indiscriminate use of these antimicrobial drugs.

*S. aureus* isolates from camels were sensitive to polymyxin B (100%), vancomycin (75%), doxycycline (75%), and nitrofurantoin (75%), in agreement with the report of Teshome et al., [77], which showed sensitivity to vancomycin (100%), doxycycline (100%), and norfloxacin (100%) in the Somali Region of Ethiopia. According to [77], there is high resistance to polymyxin B (75%) in the Somali Region, which is mainly due to prolonged and indiscriminate use of this drug in the area. However, *S. aureus* isolates were sensitive to polymyxin B (100%), since this drug is not frequently used in veterinary services in the study districts.

*S. aureus* isolates from goats were resistant to penicillin G (100%), spectinomycin (100%), polymyxin B (66.67%), and chloramphenicol (66.67%) but sensitive to doxycycline (100%), ceftriaxone (100%), vancomycin (100%), and nitrofurantoin (66.67%). A high percentage of antimicrobial resistance was observed against spectinomycin, polymyxin B, and penicillin G. These findings were in line with the results of [78], who reported 87.2% resistance to penicillin in Ethiopia.

In this study, *S. intermedius* isolates from cows were resistant to penicillin and polymyxin B but sensitive to chloramphenicol and nitrofurantoin. All *S. intermedius* isolates from camels and goats were 100% resistant to all antibiotics tested but 50% of isolates from camels were sensitive to ceftriaxone, chloramphenicol, doxycycline, and clindamycin, whereas 50% of isolates from goats were sensitive to chloramphenicol and doxycycline.

Contrary to our findings, Getahun et al. [18] reported that all three *S. intermedius* isolates from cows showed 100% susceptibility to ampicillin, penicillin, kanamycin, erythromycin, polymyxin B, streptomycin, and oxytetracycline and 75% sensitivity to sulfonamide. These authors [18] also reported the lowest proportion of erythromycin- and sulfonamide-resistant *S. aureus* isolates from dairy cows. All *S. hyicus* isolates from cows were resistant to penicillin, spectinomycin, ceftriaxone, and doxycycline. All *S. hyicus* isolates from cows were sensitive to polymyxin B, clindamycin, and nitrofurantoin. However, 66% and 33% of *S. hyicus* isolates from cows were sensitive to vancomycin and chloramphenicol, respectively. *S. hyicus* isolates from camels were resistant to penicillin, spectinomycin, ceftriazone, clindamycin, and polymyxin B and sensitive to nitrofurantoin and chloramphenicol. Fifty percent of *S. hyicus* isolates from camels were resistant to vancomycin and doxycycline. *S. hyicus* isolates from goats were resistant to penicillin, spectinomycin, and ceftriazone but showed reduced sensitivity to polymyxin B (75% sensitivity), doxycycline (75% sensitivity), and clindamycin (50% sensitivity).

Resistance to three or more antibiotic classes is multidrug resistance per the European Centre for Disease Prevention and Control (ECDC), which was found in 100% of *S. aureus* isolates from cows, camels, and goats. This result was partially in agreement with the finding Malinowski et al. [79], who reported multidrug resistance in *S. aureus* species. This indicated an alarming rise in multidrug-resistant *S. aureus* strains in cows, camels, and goats that presents a significant public health risk due to regular consumption of raw milk in this area. 

All *E. coli* isolates from cows showed reduced sensitivity (50% sensitivity) to polymyxin B and nitrofurantoin, but each was resistant to all other antibiotics tested in this study. *E. coli* isolates from goats had reduced sensitivity (50% sensitivity) to spectinomycin, vancomycin, ceftriazone and doxycycline, each and clindamycin (25% sensitivity) but were sensitive (100% sensitivity) to polymyxin B, nitrofurantoin, and chloramphenicol. Gram-negatives have an intrinsic resistance to penicillin and so *E. coli* is not sensitive to penicillin.

## 5. Conclusions

In conclusion, 100% of *S. aureus* isolates were multidrug-resistant, indicating a significant public health risk of infection from non-treatable multidrug-resistant *S. aureus* due to regular consumption of raw milk. The authors strongly recommend improving public awareness of the need to boil milk and avoid drinking raw milk at all costs through pastoral-focused agricultural extension programs. This study also indicates a need for detailed further comparative studies of *S. aureus* isolates from different niches in the area, such as from humans who had close contact with dairy animals, from human patients at clinics and hospitals in this area, and from dairy animals, as well as from the corresponding environmental surfaces to understand the transmission pathways of antimicrobial-resistant *S. aureus* among humans, animals, and their environments.

## Figures and Tables

**Table 1:** Description of the study animals and distribution of individual udder quarters or udder halves examined.

Variables	Animal Species		
	Cattle	Camel	Goat
Number of animals	60	51	60
Age (years), mean (range)	6.7 (3–15)	11.6 (6–19)	3.9 (1–8)
Number of parities, mean (range)	3.3 (1–9)	4.4 (1–9)	2.9 (1–8)
Number of quarters/halves examined	240	204	120
Number of samples by stage of lactation of animals, *n* (% of total samples in each species)	-	-	-
Early	64 (26.7)	40 (19.6)	66 (55.0)
Mid	124 (51.7)	88 (43.1)	40 (33.3)
Late	52 (21.7)	76 (37.3)	14 (11.7)
Number of herds	24	18	26
Quarters/halves examined per herd, mean (range)	9.6 (4–20)	10.2 (4–20)	4.6 (2–12)
District, *n* samples	-	-	-
Bule Hora	120	0	60
Dugda Dawa	120	204	60

**Table 2:** Distribution of variable outcomes observed among the udder quarters and udder halves.

Variable Outcomes	Camel	Cattle	Goat
Udder abnormalities, *n* (%)	-	-	-
*n*	204	240	120
0	180 (88.2)	213 (88.8)	103 (85.8)
1	2 (1.0)	1 (0.4)	6 (5.0)
2	7 (3.4)	6 (2.5)	9 (7.5)
3	10 (4.9)	12 (5.0)	0
Blind quarter/half	5 (2.5)	8 (3.3)	2 (1.7)
Milk appearance, *n* (%)	-	-	-
*n*	199	232	118
0	185 (93.0)	219 (94.4)	109 (92.4)
1	0	5 (2.2)	4 (3.4)
2	14 (7.0)	4 (1.7)	5 (4.2)
3	0	4 (1.7)	0
Clinical vs. subclinical classification, *n* (%)	-	-	-
*n*	204	240	120
Normal	170 (83.3)	191 (79.6)	94 (78.3)
Clinical	20 (9.8)	21 (8.8)	12 (10)
Subclinical	9 (4.4)	20 (8.3)	12 (10)
Nonfunctional/blind	5 (2.5)	8 (3.3)	2 (1.7)
CMT score, *n* (%)	-	-	-
*n*	199	232	118
0	170 (85.4)	191 (82.3)	94 (79.7)
1+	11 (5.5)	12 (5.2)	7 (5.9)
2+	3 (1.5)	7 (3.0)	5 (4.2)
3+	15 (7.5)	22 (9.5)	12 (10.2)

**Table 3:** Overall prevalence of udder and milk abnormalities, and prevalence of clinical mastitis by animal species.

Outcome	Camel (*n* = 199)	Cattle (*n* = 232)	Goat (*n* = 118)
Udder abnormality *	11.8 (7.3–16.2)	11.3 (7.2–15.3)	14.2 (7.9–20.4)
Milk abnormality	7.0 (3.5–10.6)	5.6 (2.6–8.6)	7.6 (2.8–12.4)
Mastitis prevalence	14.6 (9.7–19.5)	17.7 (12.8–22.6)	20.3 (13.1–27.6)
Clinical mastitis	10.1 (5.9–14.2)	9.1 (5.4–12.7)	10.2 (4.7–15.6)
Subclinical mastitis	4.5 (1.6–7.4)	8.6 (5.0–15.6)	10.2 (4.7–15.6)

* *n* = 204 (camel), 240 (cattle), and 120 (goats).

**Table 4:** Effects of stage of lactation and parity on the prevalence of udder and milk abnormalities, and clinical and subclinical mastitis in camels.

Outcomes	Predictors	Coefficient	Std. Err.	*p*-Value	95% Confidence Interval	
Udder abnormality	Stage of lactation = late	3.14	1.47	**0.033**	0.25	6.02
	Stage of lactation = mid	0.46	1.50	0.759	−2.48	3.40
	Parity	−0.14	0.25	0.574	−0.62	0.35
	Constant	−4.20	1.56	-	−7.27	−1.13
Milk abnormality	Stage of lactation = late	1.94	2.08	0.351	−2.14	6.02
	Stage of lactation = mid	−2.31	3.05	0.447	−8.28	3.66
	Parity	−0.12	0.44	0.777	−0.98	0.74
	Constant	−5.20	2.69	-	−10.47	0.06
Mastitis prevalence	Stage of lactation = late	4.22	1.70	**0.013**	0.89	7.55
	Stage of lactation = mid	1.37	1.59	0.389	−1.75	4.49
	Parity	0.47	0.21	**0.028**	0.05	0.89
	Constant	−7.10	2.00	-	−11.02	−3.17
Clinical mastitis	Stage of lactation = late	3.09	1.90	0.104	−0.64	6.82
	Stage of lactation = mid	−1.34	2.18	0.541	−5.62	2.95
	Parity	0.17	0.32	0.606	−0.46	0.80
	Constant	−5.84	2.41	-	−10.56	−1.12
Subclinical mastitis	Stage of lactation = late *	1.13	1.41	0.42	−1.62	3.89
	Stage of lactation = mid	Ref.	-	-	-	-
	Parity	0.59	0.32	0.068	−0.04	1.22
	Constant	−6.78	2.29	-	−11.27	−2.28

* Subclinical mastitis was not observed in early lactation camels, so the mid-lactating category was used as a reference. District was not assessed, since milk samples were not obtained from camels in the Bule Hora district. Std. Err.: standard error, boldface fonts indicate statistically significant observations at *p* < 0.05.

**Table 5:** Effects of stage of lactation, district, and parity on the prevalence of udder and milk abnormalities, and clinical and subclinical mastitis in cattle.

Outcomes	Predictors	Coefficient	Std. Err.	*p*-Value	95% Confidence Interval	
Udder abnormality	Stage of lactation = late	2.37	1.89	0.210	−1.34	6.09
	Stage of lactation = mid	0.96	1.40	0.495	−1.79	3.70
	District = Dugda Dawa	−2.50	1.37	0.068	−5.19	0.19
	Parity	0.12	0.28	0.670	−0.43	0.67
	Constant	−4.13	1.54	-	−7.16	−1.10
Milk abnormality #	Stage of lactation = late *	0.96	2.24	0.67	−3.44	5.35
	Stage of lactation = mid	Ref	-	-	-	-
	Parity	−0.09	0.83	0.917	−1.71	1.54
	Constant	−11.50	5.62	-	−22.52	−0.49
Mastitis prevalence	Stage of lactation = late	1.82	1.72	0.291	−1.55	5.18
	Stage of lactation = mid	0.97	1.31	0.459	−1.60	3.53
	District = Dugda Dawa	−2.70	1.27	**0.034**	−5.19	−0.21
	Parity	0.35	0.27	0.19	−0.18	0.88
	Constant	−4.15	1.52	-	−7.13	−1.18
Clinical mastitis	Stage of lactation = late	3.66	2.68	0.172	−1.59	8.91
	Stage of lactation = mid	2.56	2.15	0.233	−1.65	6.77
	District = Dugda Dawa	−1.43	1.76	0.415	−4.88	2.02
	Parity	0.17	0.32	0.586	−0.45	0.80
	Constant	−7.45	2.79	-	−12.92	−1.98
Subclinical mastitis	Stage of lactation = late	0.90	2.12	0.673	−3.26	5.06
	Stage of lactation = mid	0.49	1.61	0.761	−2.66	3.64
	District = Dugda Dawa	−3.72	2.01	0.064	−7.65	0.21
	Parity	0.35	0.37	0.345	−0.37	1.07
	Constant	−4.93	2.00	-	−8.85	−1.01

* Milk abnormalities were not observed in early lactation cattle; the mid-lactating category was used as a reference. # Milk abnormalities were not observed in Bule Hora district, so district was excluded from the analysis. Boldface fonts indicate statistically significant observations at *p* < 0.05.

**Table 6:** Effects of stage of lactation, district, and parity on the prevalence of udder and milk abnormalities, and clinical and subclinical mastitis in dairy goats.

Outcomes	Predictors	Coefficient	Std. Err.	*p*-Value	95% Confidence Interval	
Udder abnormality	Stage of lactation = late	0.23	1.39	0.871	−2.50	2.95
	Stage of lactation = mid	−0.63	1.24	0.611	−3.07	1.80
	District = Dugda Dawa	1.69	1.24	0.172	−0.74	4.12
	Parity	−0.36	0.29	0.213	−0.92	0.21
	Constant	−2.27	1.03	-	−4.28	−0.25
Milk abnormality	Stage of lactation = late	2.91	3.57	0.415	−4.09	9.90
	Stage of lactation = mid	4.35	3.31	0.189	−2.14	10.85
	District = Dugda Dawa	4.93	3.64	0.177	−2.22	12.07
	Parity	−0.68	0.83	0.416	−2.31	0.96
	Constant	−9.85	5.42	-	−20.47	0.77
Mastitis prevalence	Stage of lactation = late	0.17	2.87	0.954	−5.46	5.80
	Stage of lactation = mid	4.08	3.68	0.268	−3.13	11.29
	District = Dugda Dawa	0.04	2.29	0.985	−4.44	4.53
	Parity	0.33	0.87	0.703	−1.38	2.04
	Constant	−19.50	11.24	-	−41.53	2.53
Clinical mastitis	Stage of lactation = late	−0.12	1.97	0.95	−3.99	3.74
	Stage of lactation = mid	1.48	1.49	0.319	−1.43	4.40
	District = Dugda Dawa	3.26	1.83	0.076	−0.34	6.85
	Parity	−0.35	0.38	0.349	−1.09	0.39
	Constant	−5.07	1.91	-	−8.81	−1.33
Subclinical mastitis	Stage of lactation = late *	−	−	−	−	−
	Stage of lactation = mid	0.28	2.07	0.892	−3.78	4.34
	District = Dugda Dawa	−4.52	3.90	0.246	−12.16	3.12
	Parity	0.37	0.84	0.657	−1.28	2.02
	Constant	−15.63	8.22	-	−31.74	0.48

* No subclinical mastitis was observed for late lactation dairy goats.

**Table 7:** Bacterial species isolated from milk samples and their frequency of detection by animal species and by clinical/subclinical mastitis.

Bacteria	Clinical Mastitis			Subclinical Mastitis			Total Samples Positive for Each Bacterial Species (*n* = 64)
	Camel (*n* = 17)	Cattle (*n* = 19)	Goat (*n* = 10)	Camel (*n* = 1)	Cattle (*n* = 10)	Goat (*n* = 7)	
*E. coli*	0	2	2	0	2	0	6 (9.4)
*Staphylococcus aureus*	2	5	2	0	1	1	11 (17.2)
Coagulase-negative *Staphylococcus* species	11	6	1	0	6	1	25 (39.1)
*Staphylococcus hyicus*	2	3	2	0	0	2	9 (14.1)
*Staphylococcus intermedius*	1	1	1	1	1	1	6 (9.4)
Unidentified	1	2	0	0	0	2	5 (7.8)
Total samples positive for any bacteria	17	19	8	1	10	7	62 (96.9)

**Table 8:** Antimicrobial resistance of 32 bacterial isolates obtained from dairy animals in pastoral communities in Southern Ethiopia.

Bacterial Species	Animal Species	Number of Isolates Resistant to the Antimicrobials								
		P	S	F	PB	VAN	CRO	C	DO	DA
*S. aureus*	Cow (*n* = 6)	6	6	5	4	5	5	2	1	6
	Camel (*n* = 2)	2	2	1	0	1	2	1	1	2
	Goat (*n* = 3)	3	3	1	3	1	0	3	0	3
*S. intermedius*	Cow (*n* = 2)	2	1	0	2	1	1	0	1	1
	Camel (*n* = 2)	2	2	2	2	2	1	1	1	1
	Goat (*n* = 2)	2	2	2	2	2	2	1	1	2
*S. hyicus*	Cow (*n* = 3)	3	3	0	0	2	3	1	3	0
	Camel (*n* = 2)	2	2	0	2	1	2	0	1	2
	Goat (*n* = 4)	4	4	0	3	0	4	1	3	2
*E. coli* *	Cow (*n* = 4)	4	4	2	2	4	4	4	4	4
	Goat (*n* = 2)	2	2	0	0	2	2	0	2	1
Total (*n* = 32)		32 (100)	31 (96.9)	13 (40.6)	20 (62.5)	21 (65.6)	26 (81.3)	14 (43.8)	18 (56.3)	24 (75)

P: penicillin G; S: spectinomycin; F: nitrofurantoin; PB: polymyxin B; VAN: vancomycin; CRO: ceftriaxone; C: chloramphenicol; DO: doxycycline; DA: clindamycin. * *E. coli* was not detected in camel milk samples.

## Data Availability

All data generated in this project are included in the manuscript.
